# Alterations in shoulder kinematics are associated with shoulder pain during wheelchair propulsion sprints

**DOI:** 10.1111/sms.14200

**Published:** 2022-06-05

**Authors:** Simon J. Briley, Riemer J. K. Vegter, Victoria L. Goosey‐Tolfrey, Barry S. Mason

**Affiliations:** ^1^ Peter Harrison Centre for Disability Sport, School of Sport, Exercise and Health Sciences Loughborough University Loughborough UK; ^2^ Human Sciences Research Centre University of Derby Derby UK; ^3^ Department of Human Movement Sciences University Medical Center Groningen, University of Groningen Groningen The Netherlands

**Keywords:** shoulder pain, sports wheelchair propulsion, upper‐body kinematics, wheelchair athletes

## Abstract

The study purpose was to examine the biomechanical characteristics of sports wheelchair propulsion and determine biomechanical associations with shoulder pain in wheelchair athletes. Twenty wheelchair court‐sport athletes (age: 32 ± 11 years old) performed one submaximal propulsion trial in their sports‐specific wheelchair at 1.67 m/s for 3 min and two 10 s sprints on a dual‐roller ergometer. The Performance Corrected Wheelchair User's Shoulder Pain Index (PC‐WUSPI) assessed shoulder pain. During the acceleration phase of wheelchair sprinting, participants propelled with significantly longer push times, larger forces, and thorax flexion range of motion (ROM) than both the maximal velocity phase of sprinting and submaximal propulsion. Participants displayed significantly greater peak glenohumeral abduction and scapular internal rotation during the acceleration phase (20 ± 9° and 45 ± 7°) and maximal velocity phase (14 ± 4° and 44 ± 7°) of sprinting, compared to submaximal propulsion (12 ± 6° and 39 ± 8°). Greater shoulder pain severity was associated with larger glenohumeral abduction ROM (*r* = 0.59, *p* = 0.007) and scapular internal rotation ROM (*r* = 0.53, *p* = 0.017) during the acceleration phase of wheelchair sprinting, but with lower peak glenohumeral flexion (*r* = −0.49, *p* = 0.030), peak abduction (*r* = −0.48, *p* = 0.034), and abduction ROM (*r* = −0.44, *p* = 0.049) during the maximal velocity phase. Biomechanical characteristics of wheelchair sprinting suggest this activity imposes greater mechanical stress than submaximal propulsion. Kinematic associations with shoulder pain during acceleration are in shoulder orientations linked to a reduced subacromial space, potentially increasing tissue stress.

## 1 INTRODUCTION

Shoulder pain is a primary health concern among manual wheelchair users.^1^ This population relies on their upper limbs for all activities of daily living, such as wheelchair propulsion, which exposes their shoulders to highly repetitive stress.^2^, ^3^ Previous studies have identified associations between shoulder pain and push‐rim kinetics,^4^, ^5^, ^6^ glenohumeral and scapular kinematics,^7^, ^8^ and movement variability^7^ during manual wheelchair propulsion. Although these biomechanical features are comparable in wheelchair athletes and nonathletic wheelchair users during daily propulsion,^9^ wheelchair athletes also propel a sports‐specific wheelchair.

During wheelchair court‐sports (basketball, rugby, and tennis), athletes propel their sports wheelchair, which are configured differently to daily life chairs (i.e., higher/ lower seat height, increased camber angle).^10^ Athletes primarily propel at submaximal speeds; however, high‐speed propulsion activities, including sprints, are frequently performed.^11^, ^12^ The ability to accelerate rapidly and attain a high maximal velocity has been identified as key indicators of performance in these sports.^10^, ^13^ That said, these activities rely on the relatively small muscle mass of the upper limb and impose a large mechanical demand on the shoulder musculature, particularly the rotator cuff.^14^ Consequently, the shoulder pain risk associated with sports wheelchair propulsion may be substantial if combined with extreme upper‐limb orientations, as certain glenohumeral and scapula orientations can influence subacromial space.^2^, ^15^, ^16^ Specifically, increases in glenohumeral internal rotation, abduction and forward flexion and scapular internal rotation, downward rotation and anterior tilting are linked to reductions in subacromial space potentially inducing rotator cuff and biceps tendon stress.^16^

Previous studies have primarily characterized wheelchair sprinting in court‐sport athletes via spatio‐temporal and kinetic measures.^17^, ^18^ Evidence suggests that over the first three pushes of a wheelchair sprint athletes generate large propulsive forces to overcome the inertia of the wheelchair‐user and maximally accelerate their wheelchairs,^19^ whereas a lower magnitude but high rate‐of‐rise of applied forces is needed to maintain maximal velocity during the latter portion of the sprint.^13^, ^18^, ^20^ Given these kinetic differences, alterations in glenohumeral and scapular kinematics between the acceleration and maximal velocity phases of wheelchair sprinting may be evident. Furthermore, if the intensity of wheelchair sprinting exceeds submaximal propulsion, a larger influence on athletes' shoulder pain symptoms is to be expected. However, the specific kinematic and kinetic parameters associated with shoulder pain during these sports wheelchair propulsion tasks are unknown.

Therefore, the purpose of this study was i) to quantify and compare wheelchair propulsion biomechanics at a submaximal velocity and during the acceleration and maximal velocity phases of wheelchair sprinting in athletes' sports‐specific wheelchairs, and ii) to explore the relationship between shoulder pain and propulsion biomechanics during these activities.

## 2 MATERIALS AND METHODS

### 2.1 Participants

Twenty wheelchair athletes (age: 32 ± 11 years, body mass: 70.2 ± 11.6 kg, years of wheelchair use: 13 ± 11 years, years competing in wheelchair court‐sports: 8 ± 5 years, male = 16, female = 4) provided written informed consent and participated in this study. Ethical approval was obtained through the University's local ethics committee. All participants were full‐time manual wheelchair users that used a daily life wheelchair and a sports‐specific wheelchair which they used for regular training and competition in one of the following wheelchair court‐sports: rugby (*n* = 9), basketball (*n* = 7), and tennis (*n* = 4) (Table S1). Participants were recruited from the local community and wheelchair sports clubs through advertisements and previous study participation. Primary impairments were inclusive of spinal cord injury (SCI) C6 or below, spina bifida, and cerebral palsy, which was representative of the court‐sports at both a National/International level.

### 2.2 Shoulder pain

Shoulder pain was evaluated using the Performance‐Corrected Wheelchair User Shoulder Pain Index (PC‐WUSPI).^3^ The severity of shoulder pain was classified as mild, moderate, or severe following PC‐WUSPI thresholds described in Briley et al. (2020b^7^). Specifically, a PC‐WUSPI score of ≤51 was classified as no or mild pain, between 52.5 and 111 moderate pain, and > 112.5 severe pain. A modified upper extremity pain questionnaire (PSQ) was used to report the location (right/left) of shoulder pain.^21^

### 2.3 Experimental protocol

All trials were conducted in participants' own sports‐specific wheelchair (chair mass 12.6 ± 1.7 kg; wheel diameter 0.62 ± 0.03 m; rim diameter 0.57 ± 0.03 m; and camber angle 19 ± 1°) on a dual roller wheelchair ergometer (Lode Esseda, m988900, Groningen, Netherlands), full description provided elsewhere.^22^ The Lode Esseda ergometer simultaneously collects spatio‐temporal and kinetic parameters of wheelchair propulsion from each side which show good agreement with that of instrumented measurement wheels^22^ (Figure S1). The use of participants own sports wheelchair reflects the natural interaction between each user and their wheelchair.

Participants performed a five‐minute warm‐up involving self‐selected propulsion and dynamic stretching followed by a three‐minute submaximal propulsion trial at 1.67 m/s. The prescribed propulsion speed reflected fixed speeds used in previous daily propulsion studies of athletic populations and typical average speeds reported for wheelchair basketball match play.^12^, ^23^ Participants maintained this speed by following a visual real‐time display of the combined speed of the left and right rollers. Following a two‐minute rest period, participants performed two 10‐second sprints from a rolling start (1 m/s) 5 min apart. The 10 s sprint duration was chosen to ensure that all participants reached maximal velocity. A rolling start was used to minimize wheel slipping encountered when propelling from a stationary position on the ergometer. A trial began only when the participant reported a Rating of Perceived Exertion (RPE) score of ≤7.^24^ Verbal encouragement was provided during the sprint trials to maximize participant effort.

Upper limb kinematic data were acquired using a Vicon motion capture system (Vicon, Motion Systems Ltd. Oxford, UK) consisting of 10 cameras (MX T40‐S) recording at 200 Hz. Eighteen retroreflective markers (B&L Engineering, California, USA) were attached to anatomical landmarks of both upper limbs and the torso following the International Society of Biomechanics (ISB) recommendations.^25^ Acromion marker clusters (AMC) were used to track scapular orientation as described by Warner et al.^26^ Glenohumeral joint centers (GHJC) were determined using the Symmetrical Centre of Rotation Estimation (SCoRE) method from a bilateral circumduction trial.^27^

### 2.4 Data analysis

**FIGURE 1:**
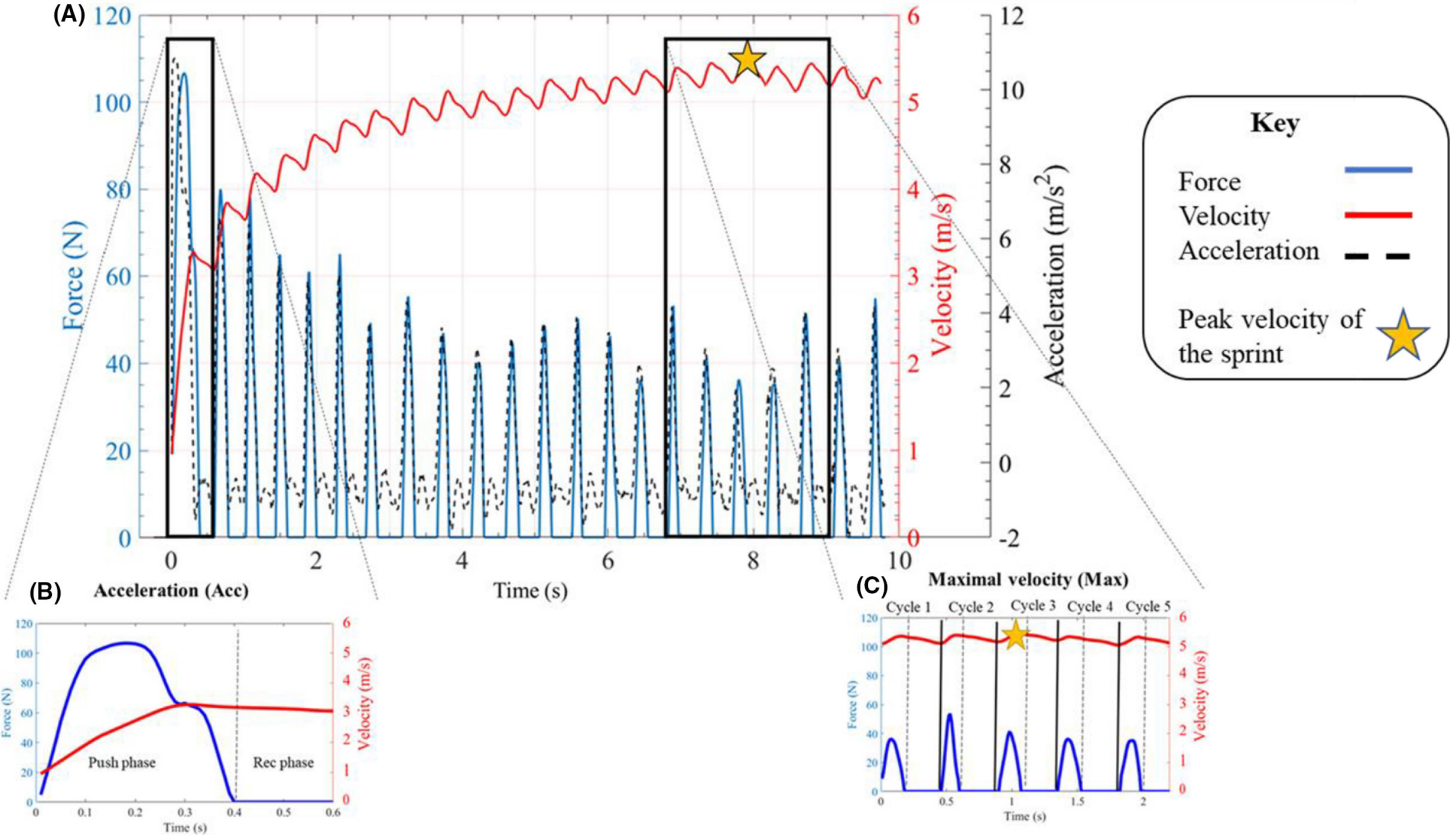
Representative force (blue), velocity (red), and acceleration (black) data of the whole 10‐second sprint (A). Highlighted view of the force and velocity of the first push—acceleration phase (B) and the maximal velocity phase (C) analyzed in this study. Rec = Recovery, Cycle = Propulsion cycle

The mean and standard deviation of kinetic and kinematic variables at each time normalized point were extracted from 10 consecutive propulsion cycles of each participant during the final 60 seconds of the submaximal propulsion trial. Two phases of the sprint were selected for analysis: acceleration and maximal velocity (Figure 1). The acceleration phase was determined as the first propulsion cycle at the onset of the sprint. Other studies have combined the first three propulsion cycles as the acceleration phase when analyzing kinetic data^18^; however, all participants in the current study displayed large intra‐individual joint kinematic differences between the first three propulsion cycles. Furthermore, the first propulsion cycle was distinctly different to the following pushes, as evidenced by a larger force output and lower inter‐individual kinematic differences. The maximal velocity phase included the propulsion cycle during which peak velocity was reached and two cycles on either side. Multiple propulsion cycles were used for the maximal velocity phase because all individuals achieved a plateau (steady‐state) and displayed comparable kinematic data across at least five propulsion cycles.

The following spatio‐temporal and kinetic parameters of wheelchair propulsion were calculated from the force and velocity data collected using the ergometer: push time, recovery time, contact angle, acceleration, force, power, and rate of rise of applied force (ROR)^9^, ^18^ (Table S2). An eighth‐order Butterworth filter with a cutoff of 10 Hz filtered force data. A fourth‐order, low‐pass Butterworth filter with a cutoff frequency of 7 Hz filtered motion analysis data. Filter cutoff frequencies were determined by residual analysis and in line with previous studies.^9^, ^28^ Euler angles of the thorax (thorax to global), glenohumeral (humerus to scapular), and scapulothoracic (scapular to thorax) were calculated.^25^ Discrete kinematic data selected for analysis were peak angles and range of motion (ROM) for thorax flexion, glenohumeral flexion, abduction and internal rotation, scapulothoracic internal rotation, downward rotation, and anterior tilt following previous studies.^5^, ^16^ For individuals with unilateral shoulder pain, the painful side was analyzed, for those with bilateral pain the most painful side was analyzed.

### 2.5 Statistical analysis

Statistical analyses were conducted in Statistical Package for Social Sciences (SPSS version 23, IBM, New York, USA). Data normality, homogeneity of variance, and sphericity were assessed by Shapiro–Wilk tests, Levene's test, and Mauchly's test of sphericity, respectively. For the within‐subject comparison to achieve a statistical power of 80% (*p* = 0.05), a minimum of 13 participants was required (G*Power, 3.1.9.2). Repeated measures ANOVA were used to determine significant main effects for propulsion condition (sub max, acceleration, maximal velocity) on the discrete kinetic and kinematic data. Where data did not satisfy the assumption of sphericity, Greenhouse–Geisser corrections were used. Significant main effects were followed by pairwise comparisons with a least‐squares difference (LSD) correction. Subsequently, statistical parametric mapping (SPM) repeated measures ANOVAs were used to examine the temporal differences in time normalized (push and recovery phases) kinetic and kinematic variables between propulsion conditions.^29^ SPM identifies regions of the push phase and recovery phase of the propulsion cycle where significant differences in the biomechanical waveforms occurred. Significant main effects were followed by post hoc SPM paired t‐tests with a Bonferroni correction (α = 0.017). The normality of biomechanical waveforms was assessed before the analysis. All SPM analyses were performed using the open‐source MATLAB code (SPM1d, v.M0.4.5, www.spm1d.org), with detailed descriptions of SPM theory and methods provided elsewhere.^29^

A one‐way independent analysis of variance (ANOVA) was used to assess the effect of wheelchair sport (basketball, rugby, tennis) and impairment type on PC‐WUSPI scores. Spearman's rank‐order correlation analyses were used to evaluate the relationship between participants' PC‐WUSPI scores and discrete spatio‐temporal, kinetic and joint kinematic parameters of wheelchair propulsion. Correlation coefficients were classified as negligible (<0.30), low (0.30 to 0.50), moderate (0.50 to 0.70), and high (>0.70) according to previous studies.^30^

## 3 RESULTS

### 3.1 Biomechanical characteristics of sports wheelchair propulsion

Peak speed attained during the first push was 1.89 ± 0.34 m/s and during the maximal velocity phase was 3.42 ± 0.66 m/s (range 2.44–4.64 m/s). During the acceleration phase of wheelchair sprinting, participants propelled with significantly longer push times, shorter recovery times, and higher stroke frequency, peak acceleration, peak force, peak ROR, and peak power compared to submaximal propulsion at 1.67 m/s (Table 1). During the maximal velocity phase, participants propelled with shorter push times and contact angles and greater stroke frequency, peak acceleration, peak power, and peak ROR than submaximal propulsion. Longer push times, contact angles and greater peak acceleration and peak force were exhibited during the acceleration phase compared to the maximal velocity phase. Peak power was significantly higher during maximal velocity phase than the acceleration phase (Table 1).

**TABLE 1:** Spatio‐temporal and kinetic comparison of submaximal propulsion (Submax) and the acceleration (Acc.) and maximal velocity (Max) phases of sprinting in participants sports‐specific wheelchairs

Variable	Sprint			ANOVA *p*	Pairwise comparisons		
	Sub. Mean (SD)	Acc. Mean (SD)	Max. Mean (SD)		Sub. ‐ Acc	Sub. – Max.	Acc ‐ Max.
Push time (s)	0.26 (0.05)	0.36 (0.09)	0.13 (0.03)	**<0.001**	**0.001**	**<0.001**	**<0.001**
Rec time (s)	0.76 (0.16)	0.32 (0.14)	0.31 (0.11)	**<0.001**	**<0.001**	**<0.001**	0.925
Stroke frequency (Hz)	1.0 (0.2)	1.5 (0.3)	2.5 (1.1)	**<0.001**	**<0.001**	**<0.001**	**<0.001**
Peak Acc (m.s^2^)	2.05 (0.98)	5.70 (3.53)	3.58 (1.43)	**<0.001**	**<0.001**	**0.004**	**0.006**
Contact ang. (°)	84.5 (23.9)	94.7 (7.6)	65.0 (7.2)	**<0.001**	0.066	**0.003**	**<0.001**
Peak force (N)	60.3 (19.8)	127.2 (56.3)	76.5 (29.9)	**<0.001**	**<0.001**	0.105	**<0.001**
Peak ROR (N/s)	1073.8 (353.8)	1706.2 (703.3)	1833.2 (795.2)	**<0.001**	**0.002**	**0.002**	0.446
Peak power (W)	101.7 (36.9)	186.2 (106.6)	272.0 (115.7)	**<0.001**	**0.002**	**<0.001**	**0.001**

*Note*: Statistical significance is indicated in bold.

Abbreviation: ROR, rate of rise of applied force.

Significantly greater peak thorax flexion, peak glenohumeral abduction and peak scapular internal rotation but lower glenohumeral flexion/extension ROM was displayed during both phases of sprinting compared to submaximal propulsion (Table 2). Thorax flexion ROM was greater during the acceleration phase than both submaximal propulsion and maximal velocity phase of sprinting. Peak thorax flexion was significantly greater during the maximal velocity phase than the acceleration phase of sprinting.

**TABLE 2:** Joint kinematic comparison of submaximal propulsion (Submax) and the acceleration (Acc) and maximal velocity (Max) phases of the sprint in participants sports‐specific wheelchairs

Variable	Sprint			ANOVA *p*	Pairwise comparisons		
	Submax. Mean (SD)	Acc. Mean (SD)	Max. velocity Mean (SD)		Sub ‐ Acc	Sub ‐ Max.	Acc ‐ Max.
Thorax flex/extension							
Peak flexion (°)	28.7(11.7)	38.4(21.7)	49.9(23.9)	**<0.001**	**0.042**	**0.001**	**0.010**
ROM (°)	12.4(6.4)	20.2(9.1)	13.8(4.2)	**0.003**	**0.008**	0.448	**0.011**
GH flex/extension							
Peak flexion (°)	32.3(15.4)	25.2(9.1)	26.5(17.3)	0.163			
ROM (°)	56.2(13.2)	47.7(10.8)	48.8(14.0)	**0.020**	**0.025**	**0.044**	0.627
GH add/abduction							
Peak abduction (°)	46.4(11.1)	52.1(15.0)	52.7(13.8)	**0.012**	**0.003**	**0.001**	0.898
ROM (°)	27.2(9.2)	30.4(8.8)	27.8(9.7)	0.390			
GH int/external rotation							
Peak int. rot. (°)	0.8(13.2)	−1.2(17.8)	−2.9(15.2)	0.543			
ROM (°)	30.4(11.5)	27.3(9.5)	29.8(9.4)	0.554			
ST int/external rotation							
Peak int. rot. (°)	39.1(8.1)	44.9(6.8)	43.5(7.4)	**0.001**	**0.004**	**0.003**	0.345
ROM (°)	23.4(5.2)	23.0(5.7)	23.6(4.7)	0.87			
ST down/up rotation							
Peak down. rot. (°)	12.7(7.5)	13.0(9.0)	11.5(8.4)	0.518			
ROM (°)	10.6(4.1)	11.9(4.7)	10.5(3.7)	0.373			
ST post/anterior tilt							
Peak ant. tilt (°)	24.4(3.9)	25.5(7.3)	26.5(6.1)	0.323			
ROM (°)	14.5(5.4)	15.2(3.6)	16.5(4.1)	0.144			

*Note*: Statistical significance is indicated in bold.

Abbreviations: GH, glenohumeral joint; ROM, range of motion; ST, scapulothoracic joint.

**FIGURE 2:**
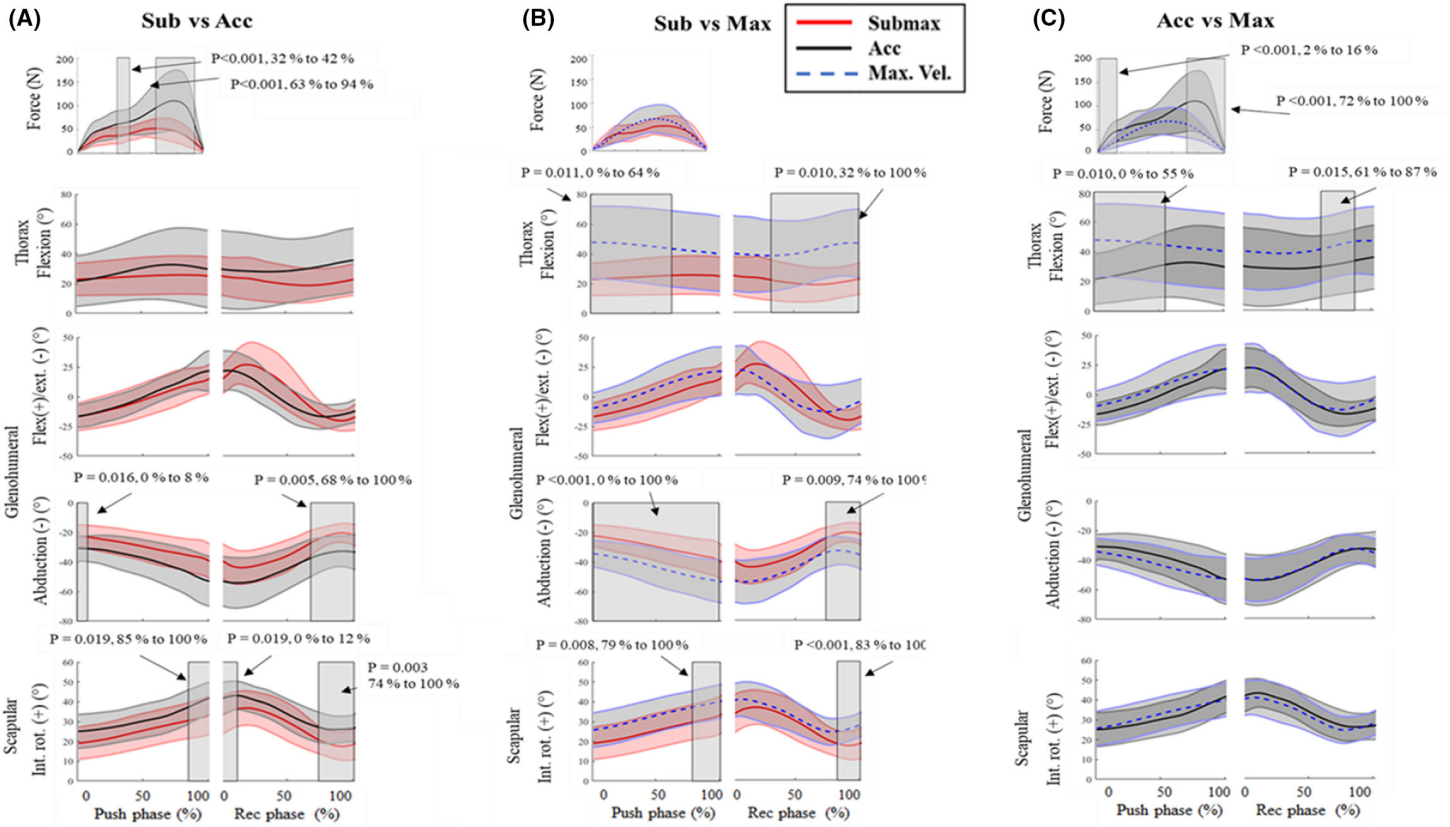
Pairwise comparisons between submaximal propulsion (Submax) and the acceleration (Acc) and maximal velocity (Max) phase of the sprint in participants sports‐specific wheelchairs using SPM post hoc tests. Mean trajectory ± SD cloud for submaximal trial (red line, red cloud), acceleration phase (black line, dark gray cloud) and maximal velocity phase (blue dashed line, light gray cloud). Rectangular shaded regions indicate significant differences between propulsion conditions with P values and percentage of push/recovery phase provided for each suprathreshold cluster

SPM repeated measures ANOVAs revealed a significant main effect for propulsion condition on force, thorax flexion, glenohumeral flexion/extension, abduction, and scapular internal rotation (Figure 2). Pairwise comparisons indicated significantly greater force during the acceleration phase of wheelchair sprinting compared to submaximal propulsion (Figure 2A). During the maximal velocity phase of sprinting, glenohumeral abduction and scapular internal rotation angles were greater than submaximal propulsion during the push phase and the recovery phase. During the maximal velocity phase of sprinting, thorax flexion, glenohumeral abduction, and scapular internal rotation were significantly greater than submaximal propulsion during the push phase and recovery phase (Figure 2B). During the acceleration phase of sprinting, participants produced significantly greater force than during the maximal velocity phase (Figure 2C). Thorax flexion was significantly greater during the maximal velocity phase than the acceleration phase during the push phase and recovery phase.

### 3.2 Relationships with shoulder pain

**FIGURE 3:**
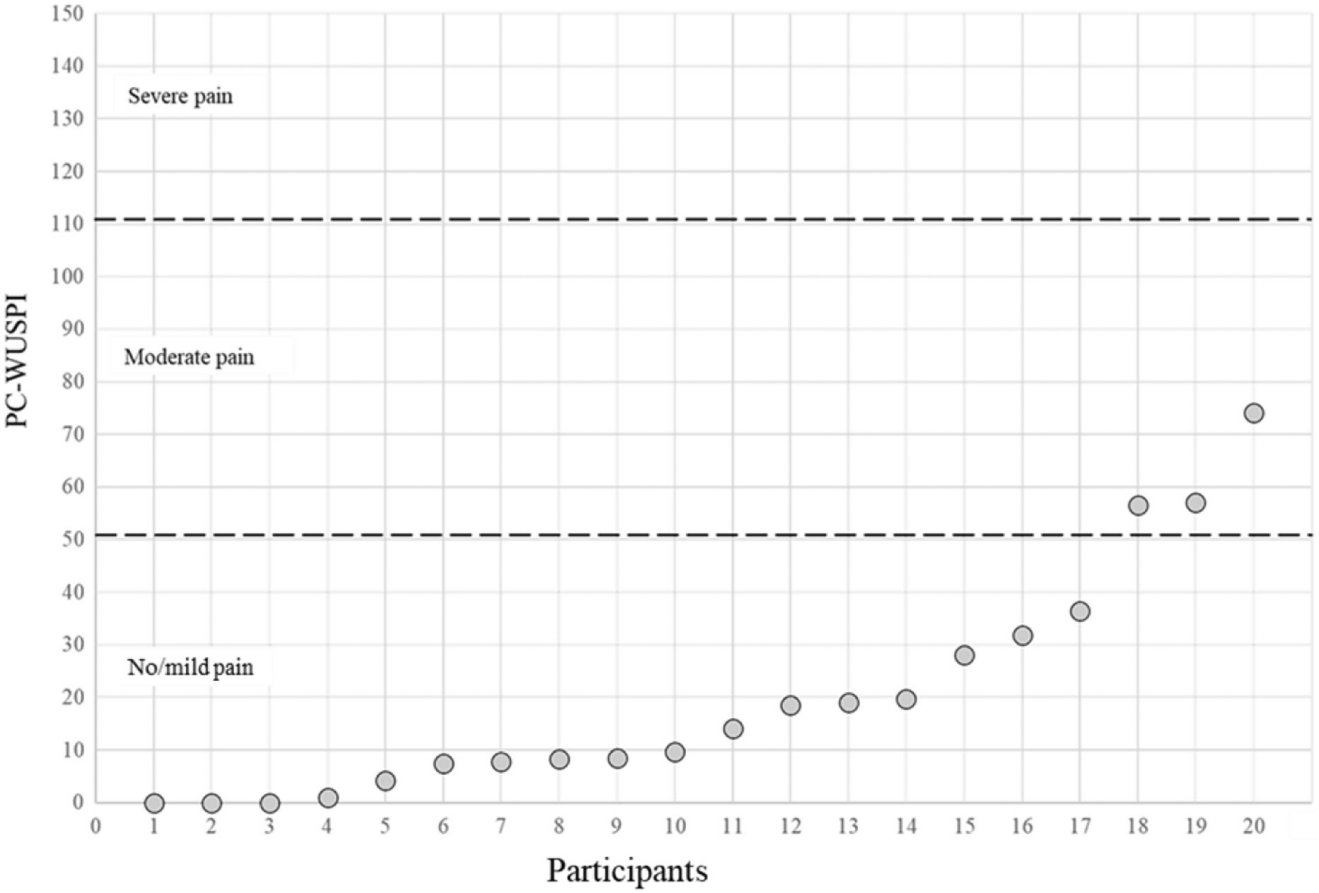
Individual PC‐WUSPI scores (gray circles) and divisions of pain groupings. Wheelchair users with no or mild pain (PC‐WUSPI ≤51) *n* = 17 and moderate pain (52.5 ≤ 111) n = 3. PC‐WUSPI = Performance‐Corrected Wheelchair User Shoulder Pain Index

Participants' mean PC‐WUSPI scores were 19.0 ± 21.4 points and ranged from zero up to 79.5 points (Figure 3). Of the 20 participants, 17 (85%) had no/mild shoulder pain and three (15%) moderate shoulder pain. No significant main effect for sport (rugby, basketball, and tennis) on PC‐WUSPI scores was observed (*F* (2, 18) = 0.24, *p* = 0.788). Additionally, no significant main effect for impairment type on shoulder pain (*F* (3, 19) = 0.06, *p* = 0.980) was observed.

Shoulder pain was not correlated with any spatio‐temporal or kinetic parameters of wheelchair propulsion (Table 3). No correlations between participants' shoulder pain severity and any kinematic parameters of submaximal sports wheelchair propulsion were observed (Table 4). Significant and moderately strong (*r* > 0.5) associations were observed between greater shoulder pain severity and greater glenohumeral abduction and greater scapular internal/external rotation ROM during the acceleration phase of wheelchair sprinting. Increasing shoulder pain severity was correlated with lower peak glenohumeral flexion and lower peak glenohumeral abduction and ROM during the maximal velocity phase of sprinting.

**TABLE 3:** Relationships between shoulder pain according to the Performance Corrected Wheelchair User Shoulder Pain Index (PC‐WUSPI) and spatio‐temporal and kinetic parameters of submaximal propulsion and the acceleration and maximal velocity phases of sprinting

Variable	Sprint					
	Submax		Acceleration		Max velocity	
	*r*	*p*	*r*	*p*	*r*	*p*
Push time (s)	0.40	0.079	−0.34	0.137	0.17	0.471
Rec time (s)	0.24	0.303	0.20	0.407	−0.06	0.793
SF (Hz)	−0.26	0.266	−0.18	0.445	0.00	0.994
Peak Acc (m.s^2^)	0.10	0.681	−0.08	0.745	0.13	0.591
Contact angle (°)	0.06	0.808	−0.32	0.170	−0.06	0.789
Peak force (N)	0.08	0.743	−0.28	0.225	0.08	0.743
Peak ROR (N/s)	0.04	0.867	−0.26	0.263	−0.05	0.825
Peak power (W)	−0.01	0.980	−0.26	0.268	−0.32	0.169

Abbreviations: Rec, recovery phase; ROR, rate of rise of applied forces; SF, stroke frequency.

**TABLE 4:** Relationships between shoulder pain according to PC‐WUSPI and joint kinematic parameters of submaximal (Submax) propulsion and the acceleration and maximal velocity phases of sprinting

Variable	Sprint					
	Submax		Acceleration		Max velocity	
	*r*	*p*	*r*	*p*	*r*	*p*
Thorax flex/extension						
Peak flexion (°)	0.05	0.835	−0.17	0.477	−0.26	0.276
ROM (°)	0.09	0.709	−0.19	0.419	−0.03	0.890
GH flex/extension						
Peak flexion (°)	−0.39	0.091	0.25	0.289	**−0.49**	**0.030**
ROM (°)	−0.32	0.167	−0.03	0.910	−0.20	0.410
GH add/abduction						
Peak abduction (°)	−0.18	0.457	0.24	0.306	**−0.48**	**0.034**
ROM (°)	−0.06	0.791	**0.59**	**0.007**	**−0.44**	**0.049**
GH int/external rotation						
Peak int. rot. (°)	−0.25	0.291	0.26	0.270	0.14	0.552
ROM (°)	0.06	0.796	0.44	0.054	−0.07	0.767
ST int/external rotation						
Peak int. rot. (°)	−0.17	0.485	0.40	0.083	−0.17	0.461
ROM (°)	−0.13	0.582	**0.53**	**0.017**	−0.06	0.811
ST Down/up rotation						
Peak down. rot. (°)	−0.31	0.190	−0.21	0.369	−0.16	0.505
ROM (°)	−0.09	0.695	0.01	0.965	−0.33	0.152
ST post/anterior tilt						
Peak ant. tilt (°)	0.40	0.081	0.38	0.103	−0.32	0.172
ROM (°)	0.06	0.811	0.00	0.995	0.19	0.430

*Note*: Statistical significance is indicated in bold.

Abbreviations: GH, glenohumeral joint; ROM, range of motion; ST, scapulothoracic joint.

## 4 DISCUSSION

The current study examined the biomechanical characteristics of sports‐specific wheelchair propulsion and associations with shoulder pain in wheelchair athletes. The biomechanical parameters of sports wheelchair propulsion differed between submaximal propulsion and during the acceleration and maximal velocity phases of wheelchair sprinting. Furthermore, greater shoulder pain severity was associated with larger glenohumeral abduction and scapular internal rotation ROM during the acceleration phase of wheelchair sprinting, but with lower peak glenohumeral flexion and abduction during maximal velocity.

The spatio‐temporal, kinetic, and joint kinematic characteristics of submaximal propulsion in a sports wheelchair were consistent with those previously observed at similar speeds in a daily wheelchair.^5^, ^7^, ^31^ Despite differences in the design and configuration of sports wheelchairs, only peak thorax flexion and glenohumeral abduction increased during sports wheelchair use compared to daily wheelchair use, with differences of ~9° and ~6°, respectively.^5^, ^9^ In addition, no associations between athletes' shoulder pain and any parameters of wheelchair propulsion biomechanics during submaximal sports propulsion were observed. Thus, the current study indicates that the propulsion biomechanics of propelling a sports wheelchair at a submaximal velocity were comparable to propelling a daily wheelchair at the same velocity.

Athletes displayed significantly greater thorax flexion, glenohumeral abduction and scapular internal rotation but lower glenohumeral flexion alongside higher peak power and ROR during the acceleration and maximal velocity phases of wheelchair sprinting compared to submaximal propulsion. The kinematic alterations to wheelchair sprinting are likely interrelated. Specifically, increased thorax flexion during push rim contact enabling reduced glenohumeral flexion but leading to greater glenohumeral abduction. Furthermore, the combination of greater thorax flexion and increased glenohumeral abduction results in a more internally rotated scapular.^32^, ^33^, ^34^ These exacerbated joint kinematic orientations may have been adopted to deliver maximal power to the push rims. However, current knowledge indicates that increases in glenohumeral abduction and scapular internal rotation reduce the subacromial space of the shoulder and greater kinetic demand elevates the mechanical stress on tissues of the shoulder.^2^, ^15^, ^16^ Therefore, the combination of joint kinematic alterations and greater kinetic demand during wheelchair sprinting may increase the acute and chronic stress to subacromial tissue imposed by wheelchair propulsion.

Biomechanical differences between phases of wheelchair sprinting were also evident. During the acceleration phase, athletes propelled their sports wheelchairs using larger contact angles, longer push times, greater force, and a larger thorax flexion range of motion compared to maximal velocity. Larger contact angles and push times facilitate the application of greater forces to the push rim, which are required to overcome the inertia of the wheelchair‐user and maximally accelerate the wheel.^20^, ^35^ In contrast, the higher wheel velocity during maximal velocity results in short contact time and contact angles and an inability to produce high push rim forces due to coupling difficulties between the hand and push‐rim.^13^, ^20^ Despite this difference in thorax kinematics, glenohumeral kinematics were comparable between the phases of wheelchair sprinting. Therefore, these kinematic differences between phases of wheelchair sprinting indicate that athletes primarily utilize flexion of the thorax rather than of the shoulder to generate high propulsive forces and larger contact angles during the initial acceleration of wheelchair sprinting.

Greater shoulder pain severity was associated with lower peak glenohumeral flexion and abduction during the maximal velocity phase of sprinting. As mentioned, these kinematic alterations are in shoulder orientations linked to subacromial tissue stress.^16^, ^35^ Given the maximal velocity phase occurred toward the end of the sprint, there may be sufficient time for shoulder pain symptoms to be evoked and individuals to respond to pain. The kinematic associations with pain reported during maximal velocity align with the protective response theory, which proposes that during tasks that may provoke painful symptoms the nervous system searches for movement patterns that are less painful by constraining motion at the painful joint/area.^36^ The low force requirement and smaller contact angles exhibited at maximal velocity may have enabled those with shoulder pain to adapt to acute pain symptoms by reducing the motion at the shoulder thereby minimizing acute pain/perceived threat of pain at the shoulder. Indeed, this alteration has been demonstrated to be associated with longitudinal increases in shoulder pain in our previous study of submaximal daily propulsion.^5^

A notable finding of the current study was that greater shoulder pain severity was associated with larger glenohumeral abduction and scapular internal rotation ROM during the acceleration phase of wheelchair sprinting. The contrasting direction of these associations with pain compared to the maximal velocity phase further highlights that any biomechanical associations with pain are specific to the constraints of the task.^37^ Specifically, individuals with greater shoulder pain may increase shoulder motions as a kinematic strategy to meet the greater force requirement of accelerating their wheelchair that is not met by an increase in thorax flexion. During the acceleration phase of wheelchair sprinting, there may be insufficient time for painful symptoms to be evoked and for individuals to respond to pain. Given the importance of initial acceleration during wheelchair sports, the kinematic associations with pain reported during acceleration may reflect a learned short‐term strategy to avoid reductions in sprint performance during this phase. Alternatively, these alterations may have been an underlying contributing factor toward individuals' current shoulder pain status. It is important to clarify that the cause and consequence cannot be stated with confidence, just assumptions based on our previous work. Nevertheless, these alterations may lead to worsening shoulder pain over time.

### 4.1 Limitations

The current study provided novel insights into sports wheelchair propulsion biomechanics and shoulder pain in court‐sport athletes. That said, time normalization of biomechanical variables within this study may have masked some temporal differences between propulsion conditions. Although the use of a roller ergometer was necessary to enable detailed comparisons of sports wheelchair propulsion conditions, its use neutralizes any steering movements that may be performed during on‐court activities. Furthermore, future work should note the large inter‐individual variability in joint kinematics present during propulsion trials. This may be explained by differences in configuration between athletes' sports wheelchairs. This study included athletes from three wheelchair sports (rugby, basketball, and tennis). Wheelchair rugby chairs typically have a lower seat height than basketball or tennis wheelchairs. Also, the impact of wheelchair athletes' primary impairment is an important consideration, particularly during sprint propulsion. To reduce this complex interaction of wheelchair configuration, athlete impairment and sports wheelchair propulsion future investigations should focus on one sport and may also categorize athletes based on impairment.

## 5 PERSPECTIVES

It may be surmised that of the propulsion conditions examined in this study, the acceleration phase of wheelchair sprinting may have the largest influence on athletes' shoulder pain symptoms. Therefore, coaches should ensure all athletes possess the physical strength and conditioning to perform this task, athletes should use a sports wheelchair which is as light as possible (if feasible) and ensure the tyres are fully inflated to reduce the kinetic demand of this task.^10^ Given the associations between shoulder pain and greater glenohumeral abduction during the acceleration phase, athletes should ensure they are appropriately fitted in their wheelchair as lower seat height and increased camber angle may increase this glenohumeral orientation.^38^ Finally, coaches should consider limiting the frequency of maximal effort sprint propulsion from a stationary or low speed start during training in individuals experiencing symptoms of shoulder pain. That said, additional research must be done to establish the feasibility of such recommendations.

## 6 CONCLUSIONS

This study revealed that the acceleration and maximal velocity phases of wheelchair sprinting coincided with a higher rate of rise of forces, greater glenohumeral abduction and scapular internal rotation than submaximal propulsion. In addition, greater shoulder pain severity was associated with increased shoulder motion during the acceleration phase of the sprint but decreased shoulder motion at maximal velocity. These findings suggest that athletes with greater shoulder pain adopt shoulder kinematic alterations during the acceleration phase of wheelchair sprinting are linked to a reductions in subacromial space, potentially leading to further tissue stress of the shoulder.

## CONFLICT OF INTEREST

There are no conflicts of interest associated with this research.

## Supporting information


**Figure S1** The Lode Esseda wheelchair ergometer used in the experimental study (a) and an example of a wheelchair secured to the ergometer using four fixed points (b).

**Table S1** Personal and sporting characteristics of the participants grouped according to wheelchair sport.
**Table S2**. Descriptions of spatio‐temporal and kinetic characteristics.

## Data Availability

The data that support the findings of this study are available on request from the corresponding author. The data are not publicly available due to privacy or ethical restrictions.
