# Supplementary material for: Alterations in shoulder kinematics are associated with shoulder pain during wheelchair propulsion sprints
Source: Scand J Med Sci Sports. 2022 Jun 5;32(8):1213–23. doi: 10.1111/sms.14200 (PMC9545165; doi:10.1111/sms.14200)
Supplement: Supplementary file 1 — Figure S1 The Lode Esseda wheelchair ergometer used in the experimental study (a) and an example of a wheelchair secured to the ergometer using four fixed points (b). [file SMS-32-1213-s002.docx]

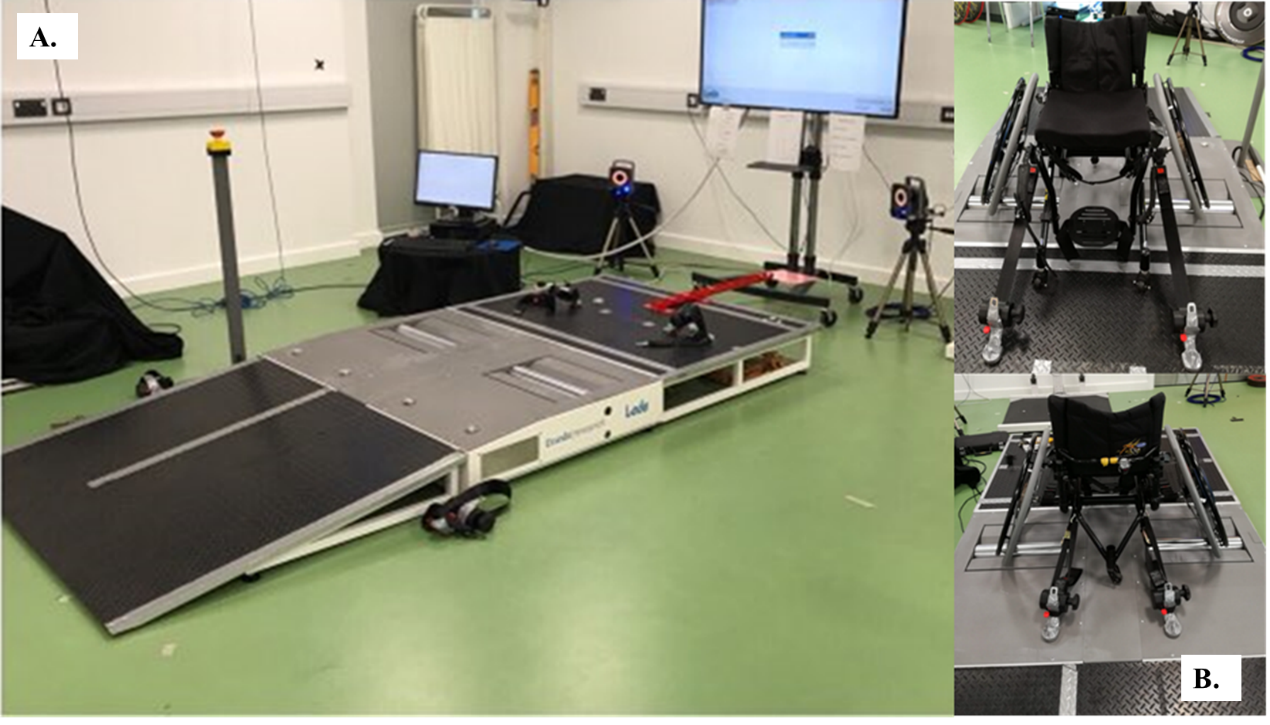
**Supplemental Figure 1**. The Lode Esseda wheelchair ergometer used in the experimental study (a) and an example of a wheelchair secured to the ergometer using four fixed points (b).
