# Supplementary material for: Alterations in shoulder kinematics are associated with shoulder pain during wheelchair propulsion sprints
Source: Scand J Med Sci Sports. 2022 Jun 5;32(8):1213–23. doi: 10.1111/sms.14200 (PMC9545165; doi:10.1111/sms.14200)
Supplement: Supplementary file 2 — Table S1 Personal and sporting characteristics of the participants grouped according to wheelchair sport. Table S2. Descriptions of spatio‐temporal and kinetic characteristics. [file SMS-32-1213-s001.docx]

**Supplemental Table 1.** Personal and sporting characteristics of the participants grouped according to wheelchair sport.

|  | WCR (n = 9) | WCB (n = 7) | WCT (n = 4) |
| --- | --- | --- | --- |
| Age (Yr) | 31(11) | 33(12) | 33(11) |
| Years as MWU (Yr) | 14(9) | 12(17) | 12(7) |
| Sex (m/f) | 7/2 | 5/2 | 4/0 |
| Body mass (kg) | 69.1(15.5) | 69.7(8.2) | 71.0(4.4) |
| Impairment | |  |  |
| SCI (C/T) | 5 | 3 | 2 |
| CP | 2 | 2 | 1 |
| SB | 0 | 0 | 0 |
| Other | 2 | 2 | 1 |
| Sports |  |  |  |
| Years competing | 8(4) | 8(6) | 8(4) |
| Classification | 1.6(0.5) | 2.9(1.1) | 2 (Open)/ 2 (quad) |

Note: SCI = Spinal cord injury, CP = Cerebral Palsy, SB = Spina bifida. WCR = Wheelchair rugby, WCB = Wheelchair basketball, WCT = Wheelchair tennis.

**Supplemental Table 2.** Descriptions of spatiotemporal and kinetic characteristics.

| Variable | Description |
| --- | --- |
| SF (push/min) | Number of pushes completed per minute |
| Push time (s) | The duration of hand push rim contact |
| Recovery time (s) | Duration of non push rim contact |
| Contact angle (°) | Wheel rotation angle during hand push rim contact |
| Peak force (N) | Peak force applied to ergometer roller |
| Peak power (W) | Torque multiplied by angular velocity |
| Rate of rise of applied force (ROR) | The peak of the derivate of force |

Note: SF=Stroke frequency
